# A priori and a posteriori dietary patterns at the age of 1 year and body composition at the age of 6 years: the Generation R Study

**DOI:** 10.1007/s10654-016-0179-x

**Published:** 2016-07-06

**Authors:** Trudy Voortman, Elisabeth T. M. Leermakers, Oscar H. Franco, Vincent W. V. Jaddoe, Henriette A. Moll, Albert Hofman, Edith H. van den Hooven, Jessica C. Kiefte-de Jong

**Affiliations:** 1 The Generation R Study Group, Erasmus MC, University Medical Center, Rotterdam, the Netherlands; 2 Department of Epidemiology, Erasmus MC, University Medical Center, Rotterdam, the Netherlands; 3 Department of Pediatrics, Erasmus MC, University Medical Center, Rotterdam, the Netherlands; 4 Leiden University College, The Hague, the Netherlands

**Keywords:** Diet, Dietary patterns, Fat mass, Fat-free mass, Obesity, Children

## Abstract

Dietary patterns have been linked to obesity in adults, however, not much is known about this association in early childhood. We examined associations of different types of dietary patterns in 1-year-old children with body composition at school age in 2026 children participating in a population-based cohort study. Dietary intake at the age of 1 year was assessed with a food-frequency questionnaire. At the children’s age of 6 years we measured their body composition with dual-energy X-ray absorptiometry and we calculated body mass index, fat mass index (FMI), and fat-free mass index (FFMI). Three dietary pattern approaches were used: (1) An a priori-defined diet quality score; (2) dietary patterns based on variation in food intake, derived from principal-component-analysis (PCA); and (3) dietary patterns based on variations in FMI and FFMI, derived with reduced-rank-regression (RRR). Both the a priori-defined diet score and a ‘Health-conscious’ PCA-pattern were characterized by a high intake of fruit, vegetables, grains, and vegetable oils, and, after adjustment for confounders, children with higher adherence to these patterns had a higher FFMI at 6 years [0.19 SD (95 % CI 0.08;0.30) per SD increase in diet score], but had no different FMI. One of the two RRR-patterns was also positively associated with FFMI and was characterized by intake of whole grains, pasta and rice, and vegetable oils. Our results suggest that different a priori- and a posteriori-derived health-conscious dietary patterns in early childhood are associated with a higher fat-free mass, but not with fat mass, in later childhood.

**Electronic supplementary material:**

The online version of this article (doi:10.1007/s10654-016-0179-x) contains supplementary material, which is available to authorized users.

## Introduction

Childhood adiposity is of great concern because of its adverse consequences for both short and long term health [1]. Diet in early childhood may be an important target for prevention of childhood obesity, but nevertheless, there are not many studies that examined overall diet of preschool children in relation to later body composition [2, 3]. The few studies that were performed in children, mostly in school-age children, reported associations between higher scores on a ‘Snacking’ dietary pattern, or on a pattern characterized by high fat and low fiber intake and a higher risk for obesity [4, 5], and similarly, between higher diet quality scores and a lower risk for obesity [6, 7].

These studies used different methods to identify these dietary patterns. The latter studies, for example, assessed diet quality using an a priori-approach on the basis of dietary guidelines [6, 7], whereas the other studies used an a posteriori-approach based on variation in dietary intake of the study population [4, 5]. Another a posteriori-approach is to construct dietary patterns based on the variation in specific markers related to health [8]. These analyses are used to identify patterns that best predict variation in for example nutrient intakes or biomarkers [5, 9], but can also be used to predict variation in body composition [10]. Consequently, the results indicate which combination of foods and beverages best predict body composition, but this approach in itself does not take into account non-dietary confounding variables and thus does not give information on whether associations persist after adjustment for sociodemographic or lifestyle factors. Although these approaches are based on different methods, they can all help to identify healthy or unhealthy dietary patterns and can form the starting point for development of new dietary guidelines [11].

Studies on dietary patterns in relation to obesity among young children are scarce [2]. Furthermore, many studies only used body mass index (BMI) as measure of obesity, whereas a higher fat mass is associated with poor cardiometabolic health [12] and a higher lean mass has been associated with improved cardiovascular and metabolic health [13–15]. This highlights the need for studies on dietary patterns in relation to specific body composition measures.

Therefore, we explored the associations between dietary patterns in children at the age of 1 year and fat mass index (FMI), and fat-free mass index (FFMI) at the age of 6 years. We applied three different approaches for dietary patterns: (1) an a priori-defined diet quality score based on dietary guidelines for preschool children; (2) a posteriori-derived dietary patterns based on variations in food intake, extracted using principal component analysis; and (3) a posteriori-defined patterns based on variations in body composition outcomes (FMI and FFMI), identified using reduced-rank regression.

## Methods

### Study design and population

**Fig. 1:**
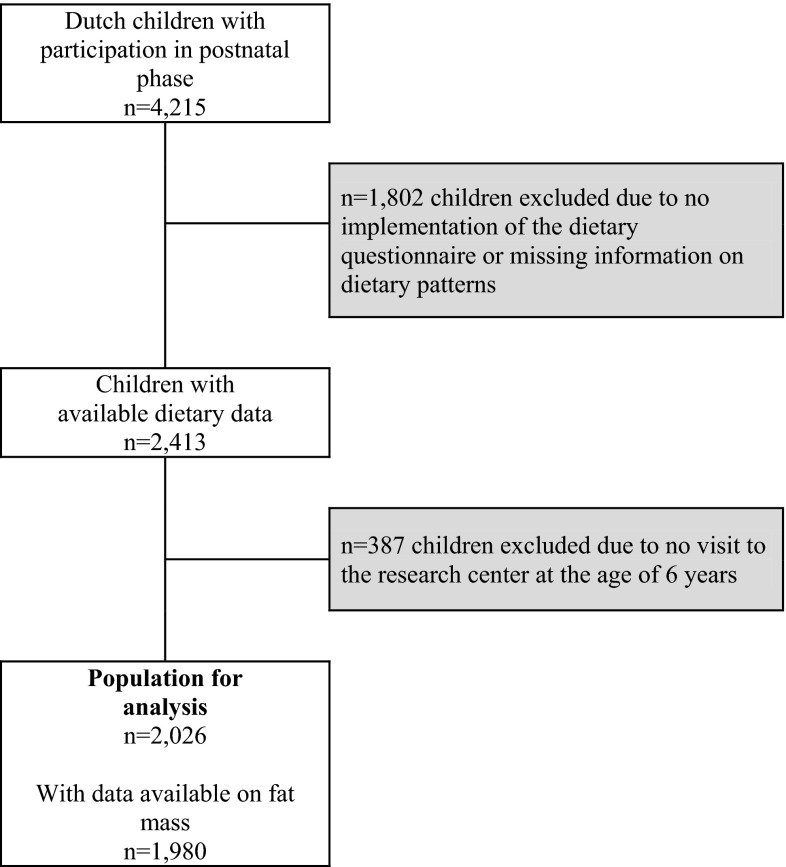
Flow chart of study participants included for the main analysis

This study was embedded in the Generation R Study, a population-based prospective cohort study among mothers and their children from fetal life onward in Rotterdam, the Netherlands [16]. All pregnant women with an expected delivery date between April 2002 and January 2006 living in the area of Rotterdam were eligible and a total of 9778 women were enrolled. The response rate based on the number of children at birth was 61 %. In total, 7893 children were available for follow-up after birth and 6690 children participated in the follow-up measurements at the age of 6 years [16]. The study was conducted according to the guidelines of the Helsinki Declaration and approved by the Medical Ethics Committee of Erasmus Medical Center, Rotterdam. All parents provided written informed consent. Further details of the cohort are described elsewhere [16]. To avoid the influence of cultural differences in dietary patterns, our analyses were restricted to children with a Dutch ethnicity [17]. Of the 7893 children available for preschool follow-up, 4215 had a Dutch ethnic background. Data on dietary patterns were available for 2413 of them [18]. At the age of 6 years, 2026 (84 %) of these children visited the research center and had anthropometrics measured, and body fat measures were available in 1980 of them (Fig. 1).

### Dietary intake

Dietary intake was assessed at a median age of 12.9 months (95 % range 12.2–19.2) using a 211-item semi-quantitative food-frequency questionnaire (FFQ), which included foods that are frequently consumed by Dutch children between 9 and 18 months of age [18, 19]. The FFQ included questions on the frequency of consumption, amounts, and types of food items and on preparation methods. Portion sizes in grams day were estimated using standardized household measures. Total energy and nutrient intake was calculated using the Dutch Food Composition Table (NEVO) of 2006. The FFQ was validated against three 24 h-recalls in a representative sample of 32 Dutch children. Interclass correlation coefficients for nutrient intakes ranged from 0.4 to 0.7 [18, 19].

### Dietary patterns

An a priori dietary pattern was defined using a previously developed diet quality score for preschool children [19]. This score was developed using international dietary guidelines as a basis and includes intake of the following ten food groups: high intake of vegetables; fruit; bread and cereals; rice, pasta, potatoes, and legumes; dairy; meat, poultry, eggs and meat substitutes; fish; and fats and oils; and low intake of candy and snacks; and sugar-sweetened beverages (Table 1 and [19]). The score ranges from 0 to 10 on a continuous scale, with a higher score representing a healthier diet. The diet score was standardized to a recommended energy intake for 1–3-year-old children of 1200 kcal/d [19].

**Table 1:** Food groups included in the dietary patterns

	Positive (+) or negative (−) score	Factor loadings from PCA^b,c^ (after varimax rotation)		Factor loadings from RRR^c^	
	Diet quality score^a^	‘Health-conscious’ pattern	‘Western-like’ pattern	RRR pattern 1	RRR pattern 2
Refined cereals	Not included		0.57	0.22	
Whole cereals	+				0.41
Pasta and rice	+	0.62			0.46
Dairy	+				0.27
Fruit	+	0.32			0.28
Soya substitutes	Not included				
Vegetables	+	0.74		0.40	0.38
Potatoes	+	0.61		0.34	
Soups and sauces	Not included		0.23	0.44	
Savory snacks	−		0.59		
Confections	−		0.72		
Vegetable oils	+	0.50			0.37
Other fats	+		0.58		0.20
Fish	+	0.22		0.42	
Shellfish	+				
Meat	+	0.21	0.27	0.30	
Eggs	+				
Legumes	+	0.59			
Sugar-containing beverages	−		0.59	0.30	
Non-sugar-containing beverages	Not included				0.28
Composite dishes	Not included				
Explained variation (%) in food group intake		16.3	8.2	5.7	12.5
Explained variation (%) in FMI and FFMI		0.4	0.1	1.8	0.8

^a^Further details in Voortman et al. [19]

^b^Further details Kiefte-de Jong et al. [18]

^c^Only factor loadings ≥|0.2| are reported

A posteriori dietary patterns on the basis of variation in food group intake were extracted using principal component analysis (PCA), that explained the maximum variation in the intake of 21 food groups (Table 1 and [18]). To reduce correlation between the factors, a Varimax rotation was used. Only dietary patterns with an Eigenvalue of ≥1.5 were extracted [18].

A posteriori dietary patterns on the basis of variation in FMI and FFMI were identified using reduced-rank regression (RRR), in order to identify dietary patterns that best predict child body composition [20]. For this method, we used age- and sex-adjusted FMI and FFMI as response variables and we used the same 21 food groups (Table 1) as used in the PCA as predictor variables.

### Body composition

Children’s anthropometrics and body composition were measured by well-trained staff at a median age of 5.9 years (95 % range 5.7–6.5) in a dedicated research center in the Sophia Children’s Hospital in Rotterdam. Height was determined in standing position to the nearest millimeter without shoes with a Harpenden stadiometer (Holtain Limited, Dyfed, U.K.). Weight was measured using a mechanical personal scale (SECA, Almere, the Netherlands) and body mass index (BMI) was calculated (body weight (kg)/height (m)^2^).

Total body, android, and gynoid fat mass were measured using a Dual-energy X-ray absorptiometry (DXA) scanner (iDXA, GE-Lunar, 2008, Madison, WI, USA), which analyzed fat, lean and bone mass of the total body and specific regions using enCORE software v.13.6. We calculated fat mass index (FMI) [fat mass (kg)/height (m)^2^] and fat-free mass index (FFMI) [fat-free mass (kg)/height (m)^2^] [21]. As secondary outcome measures we also examined android/gynoid ratio (android fat mass divided by gynoid fat mass); and body fat percentage (BF %) (fat mass as percentage of total body weight). We calculated age- and sex-specific SD scores for all outcomes based on the total Generation R Study sample with body composition measurements available the age of 6 years (*n* = 6491*)*.

### Covariates

Information on maternal age, parity, folic acid supplement use, paternal education, paternal smoking and household income were obtained using questionnaires at enrolment in the study [16]. Educational level and household income were categorized into three groups according to Dutch standard classifications [22]. Maternal smoking and alcohol use during pregnancy were assessed using questionnaires in each trimester and was categorized into never; until pregnancy was known; or continued during pregnancy [23]. Maternal anthropometrics were measured at enrolment at the research center, without shoes and heavy clothing [16].

Information on breastfeeding was obtained from delivery reports and postnatal questionnaires and was categorized as never breastfeeding; any partial breastfeeding in the first 4 months of life and; full breastfeeding in the first 4 months of life [18]. Timing of introduction of complementary feeding in the first year of life was assessed using the FFQ at 1 year and categorized into three groups: <3 months, 3–6 months, or ≥6 months [24]. Child’s height and weight around the age of 1 year were measured around at the Community Child Health Centers and BMI SD-scores were calculated using Dutch reference curves [25]. Information about television watching at the age of 2 years (h/d), as an indicator of sedentary behavior, was assessed with a questionnaire.

### Statistical analyses

All dietary patterns were expressed in SD scores to facilitate comparability of the results. The dietary pattern scores were analyzed as continuous variables and also categorized into quartiles with the first quartile as reference. A higher SD score represents a higher adherence to the dietary pattern. All body composition outcomes were expressed as age- and sex-adjusted SD scores and analyzed as continuous variables.

We used multivariable linear regression models to assess the associations between the five dietary patterns and each of the body composition measures in separate models. All models were adjusted for energy intake to reduce extraneous variation and adjust for confounding by energy intake [26]. Crude models were adjusted for child sex, age at dietary assessment, and total energy intake (model 1). The multivariable models were further adjusted for maternal age, BMI at enrolment, parity, folic acid supplement use, and smoking and alcohol use during pregnancy; paternal smoking and education; household income; child breastfeeding in the first 4 months of life, timing of introduction of complementary feeding, and television watching at the age of 2 years. These covariates were selected as potential confounders because they were associated with at least one of the dietary patterns in our study [18, 19]. As sensitivity analysis, we additionally adjusted the multivariable models for BMI (SDS) at the age of 1 year to assess whether dietary patterns at age 1 year were associated with body composition at 6 years independent of BMI at baseline. We also performed sensitivity analyses from which we excluded children who still received breast milk or a substantial amount of infant formula (i.e., more than 500 kcal/d) (n = 386) at the time of dietary assessment. To examine whether associations of the dietary patterns with height did not create spurious relations with FMI and FFMI [27], we additionally examined the association of absolute fat mass (kg) and fat-free mass (kg) independent of height.

To reduce potential bias associated with missing data, missing values of covariates were multiple imputed (*n* = 10 imputations) using the Fully Conditional Specification method (predictive mean matching), assuming no monotone missing pattern [28]. Because we previously identified differences in associations of diet with body composition between boys and girls [29, 30], we evaluated the statistical interaction with sex by adding the product term of child sex and each of the dietary patterns to the multivariable models on FMI and FFMI. Statistical analyses were performed using SPSS version 21.0 (SPSS Inc., Chicago, IL, USA) and SAS version 9.1 (SAS Institute, Cary, NC, USA).

## Results

### Subject characteristics

Characteristics of the children and their parents are presented in Table 2. Most of the women were nulliparous at enrolment in the study (63.0 %) and did not smoke during pregnancy (79.5 %); and most of the children received breastfeeding exclusively (30.2 %) or partially (60.1 %) in their first 4 months of life (Table 2). Subject characteristics before and after the multiple imputation procedure are presented in Online Resource 1. At the age of 6 years, median (95 % range) body fat percentage was 23.1 % (16.2–34.3) and median BMI was 15.7 kg/m^2^ (13.6–19.1).

**Table 2:** Characteristics of the children and their parents (n = 2026)

	n	Median (95 % range) or percentage^a^
*Parental characteristics*		
Maternal age (year)	2026	32.3 (22.9–39.9)
Maternal BMI at enrollment (kg/m^2^)	1849	23.3 (18.9–34.8)
Parity		
0	1242	63.0 %
1	557	28.2 %
≥2	173	8.8 %
Folic acid supplement use (%)		
Started periconceptional	970	63.5 %
Started in first 10 weeks	448	29.3 %
Never	110	7.2 %
Alcohol use during pregnancy (%)		
Never	517	30.8 %
Until pregnancy was known	278	16.6 %
Continued	881	52.6 %
Smoking during pregnancy (%)		
Never	1466	79.5 %
Until pregnancy was known	186	10.1 %
Continued	193	10.5 %
Paternal smoking (%)		
No	947	61.7 %
Yes	589	38.3 %
Paternal education (%)		
Primary or secondary school	542	29.0 %
Higher education	1328	71.0 %
Net household income per month (%)		
<2200 €	359	20.3 %
≥2200 €	1413	79.7 %
*Child characteristics*		
Gender (%)		
Boys	1002	49.5 %
Girls	1024	50.5 %
Breastfeeding (%)		
Exclusive for at least 4 months	552	30.2 %
Partial in the first 4 months	1101	60.2 %
Never	176	9.6 %
Introduction complementary feeding (%)		
After 6 months	800	39.6 %
3–6 months	1136	56.3 %
0–3 months	83	4.1 %
Children receiving breast milk or ≥500 kcal/d from infant formula at age of dietary assessment (%)		
Yes	386	19.0 %
No	1640	81.0 %
Television watching at 2 years (h/day)	1915	0.9 (0–2)
Age at FFQ (mo)	2026	12.9 (12.2–19.2)
Total energy intake (kcal/d)	2026	1267 (737–2080)
Age at center visit (y)	2026	5.9 (5.7–6.5)
Height at center visit (cm)	2026	118.4 ± 5.0
Weight at center visit (kg)	2026	22.2 ± 3.0
Body mass index at center visit (kg/m^2^)	2026	15.8 ± 1.4
Fat-free mass index at center visit (kg/m^2^)	1980	11.9 ± 0.8
Fat mass index at center visit (kg/m^2^)	1980	3.7 ± 1.0
Body fat percentage at center visit	1980	23.6 ± 4.5
Android/gynoid fat ratio at center visit	1980	0.24 ± 0.05

^a^Values are valid percentages for categorical variables or medians (95 % range) or means ± SDs for continuous variables on the basis of unimputed data in a total sample of 2026

### Dietary patterns

Mean (±SD) diet quality score at the age of 1 year was 4.2 (±1.3) on a theoretical range of 0–10. With PCA, we identified two dietary patterns in our study population, which we named: a ‘Health-conscious’ dietary pattern, as it was characterized by high intake of fruits, vegetables, oils, legumes, pasta, and fish; and a ‘Western-like’ dietary pattern, that was characterized by high intake of snacks, animal fats, refined grains, confectionery and sugar-containing beverages (Table 1 and [18]) . With RRR, two dietary patterns were extracted that explained the maximal variance in FMI and FFMI. The first RRR pattern was positively correlated with both FMI and FFMI, and was characterized by high intake of refined grains, meat, potatoes, fish, soups and sauces, and sugar-containing beverages (Table 1). The second RRR pattern was positively correlated with FFMI, but inversely with FMI. This pattern was characterized by high intake of whole grains, pasta and rice, dairy, fruit, vegetable oils and fats, and non-sugar-containing beverages (Table 1).

### Dietary patterns and body composition

In multivariable adjusted models, a higher adherence to the PCA-derived ‘Health-conscious’ dietary pattern or a higher diet quality score at the age of 1 year was associated with a higher FFMI at the age of 6 years (Table 3). Children in the highest quartile of the diet score had a 0.19 SD higher FFMI (95 % CI 0.08; 0.30) than children in the lowest quartile. These patterns were not associated with FMI (Table 3), or with BF % or with android/gynoid ratio (Online Resource 2). Adherence to the PCA-derived ‘Western’ dietary pattern at the age of 1 year was not consistently associated with any of the body composition measures the age of 6 years.

**Table 3:** Multivariable associations of dietary patterns at 1 year of age with childhood body composition at 6 years of age

	FMI (SDS) *n* = 1980 β (95 % CI)	FFMI (SDS) *n* = 1980 β (95 % CI)
Diet quality score		
Per SD	0.02 (−0.01; 0.05)	0.06 (0.02; 0.10)**
Q1 (low adherence)	*Reference*	*Reference*
Q2	0.03 (−0.06; 0.11)	0.09 (−0.02; 0.20)
Q3	−0.01 (−0.09; 0.08)	0.14 (0.02; 0.25)*
Q4 (high adherence)	0.07 (−0.01; 0.16)	0.19 (0.08; 0.30)**
Health-conscious pattern (PCA)		
Per SD	0.01 (−0.03; 0.04)	0.05 (0.01; 0.09)*
Q1 (low adherence)	*Reference*	*Reference*
Q2	0.02 (−0.07; 0.10)	0.02 (−0.09; 0.13)
Q3	0.03 (−0.05; 0.12)	0.13 (0.02; 0.24)*
Q4 (high adherence)	0.04 (−0.05; 0.13)	0.17 (0.06; 0.29)**
Western pattern (PCA)		
Per SD	−0.01 (−0.05; 0.03)	0.02 (−0.04; 0.07)
Q1 (low adherence)	*Reference*	*Reference*
Q2	0.02 (−0.06; 0.11)	−0.01 (−0.12; 0.10)
Q3	0.06 (−0.03; 0.15)	0.15 (0.04; 0.27)**
Q4 (high adherence)	−0.01 (−0.11; 0.09)	0.09 (−0.04; 0.22)
RRR pattern 1		
Per SD	0.10 (0.06; 0.13)**	0.09 (0.04; 0.14)**
Q1 (low adherence)	*Reference*	*Reference*
Q2	0.10 (0.01; 0.18)*	0.08 (−0.03; 0.19)
Q3	0.09 (0.01; 0.18)*	0.07 (−0.04; 0.18)
Q4 (high adherence)	0.18 (0.10; 0.27)**	0.23 (0.11; 0.35)**
RRR pattern 2		
Per SD	−0.03 (−0.07; 0.00)	0.07 (0.02; 0.11)**
Q1 (low adherence)	*Reference*	*Reference*
Q2	−0.06 (−0.15; 0.02)	−0.02 (−0.13; 0.10)
Q3	−0.01 (−0.10; 0.08)	0.18 (0.06; 0.29)**
Q4 (high adherence)	−0.07 (−0.17; 0.03)	0.19 (0.06; 0.32)**

Values are regression coefficients (95 % CI) that reflect the difference in outcome (age- and sex-adjusted SD scores) per 1 SD increase in exposure and for quartiles of exposure compared to the lowest quartile, based on imputed data

Models are adjusted for maternal age, BMI at enrollment, parity, folic acid supplement use, smoking and alcohol use during pregnancy; paternal smoking and education; household income; and child sex, breastfeeding in the first 4 months of life, timing of introduction of complementary feeding, age at dietary measurement, total energy intake at 1 year, and television watching at age 2 years

Bold values indicate statistically significant effect estimates (*p* < 0.05)

*FMI* fat mass index, *FFMI* fat-free mass index, *PCA* principal component analysis, *RRR* reduced rank regression

* *p* < 0.05; ** *p* < 0.01

The first RRR-derived pattern, which was positively correlated with FMI and FFMI, remained positively associated with both FMI and FFMI after adjustment for confounders (Table 3) and was also associated with a higher BF% and a higher android/gynoid ratio (difference in BF% 0.14 (95 % CI 0.04; 0.24) SD for highest vs. lowest quartile) (Online Resource 2). The second RRR-pattern, which was positively correlated with FFMI and inversely correlated with FMI, remained positively associated with FFMI (0.19 (95 % CI 0.06; 0.32) SD for highest vs. lowest quartile) after adjustment, but was no longer significantly associated with FMI (Table 3). However, this second RRR-pattern remained associated with a lower BF % and a lower android/gynoid ratio in the multivariable model [−0.12 (95 % CI −0.23; −0.02) SD in BF % for highest vs. lowest quartile] (Online Resource 2).

### Additional analyses

Additional adjustment for BMI-for-age at 1 year only slightly attenuated the effect estimates and all associations with FFMI remained significant (Online Resource 3). The inverse association between the second RRR-pattern and FMI that disappeared after adjustment for the confounders in our main models became statistically significant again in the model with adjustment for BMI-for-age at 1 year. Exclusion of children who still received breastfeeding or a substantial amount of infant formula (n = 386) did not affect the associations of the diet score or the RRR patterns with body composition, but the association of the ‘Health-conscious’ dietary pattern with FFMI slightly attenuated and was no longer statistically significant (Online Resource 4). Associations of the dietary patterns with absolute fat mass and fat-free mass with and without adjustment for height were similar to associations with FMI and FFMI (data not shown). We observed no significant interactions of the dietary patterns with child sex on any of the outcomes.

## Discussion

In a large population-based cohort study in young children, we observed that higher adherence to an a priori-defined diet quality score or to an a posteriori-defined ‘Health-conscious’ dietary pattern at the age of 1 year was associated with a higher FFMI, but not with FMI at the age of 6 years. Using reduced-rank regression, we additionally identified dietary patterns that predict child body composition. A pattern that was associated with a higher FFMI, but not with FMI, was characterized by high intake of whole grains, pasta and rice, dairy, fruit, vegetable oils and fats, and non-sugar-containing beverages. Additionally, a pattern positively associated with both FMI and FFMI was identified, which was characterized by high intake of refined grains, meat, potatoes, fish, soups and sauces, and sugar-containing beverages. These associations were all independent of total energy intake and parental and child sociodemographic and lifestyle factors.

### Interpretation and comparison with previous studies

We observed small but statistically significant positive associations between better a priori defined diet quality or higher scores on PCA- or RRR-derived health-conscious dietary patterns in early childhood and subsequent FFMI, but not with FMI. These three patterns were all characterized by high intake of foods generally considered to be healthy (vegetables, fruit, whole grains, and vegetable oils). The associations with a higher FFMI suggest that these dietary patterns can be beneficial for health in later life, as higher lean mass is associated with improved cardiovascular and metabolic health [13–15].

Three previous prospective studies examined the association of overall diet in early childhood with body composition later in life [31–33], of which only one separately assessed fat and fat-free mass [33]. In line with our results, this latter study among 536 children in the U.K. observed that a higher adherence to a PCA-derived ‘infant guidelines’ dietary pattern at the age of 12 months was associated with higher lean mass index but not FMI at the age of 4 years [33]. This pattern was characterized by a high intake of fruit, vegetables, cooked meat and fish, and rice and pasta; and low intake of commercial baby foods [34]. Other dietary patterns were not examined. The other two studies both assessed predefined diet quality on the basis of dietary guidelines. Adherence to the ‘Raine Eating Assessment in Toddlers’ index at 1–3 years of age in 2562 Australian children was not consistently associated with BMI during childhood and adolescence [32]. In a large cohort of U.K. children (*n* = 4798), a higher score on a ‘Complementary Feeding Utility Index’ at the age of 6 months was also not associated with BMI at the age of 7 years after adjustment for sociodemographic variables, but was associated with a lower waist circumference [31]. Our findings, together with the findings from the U.K. study [33] suggest that not total body weight or BMI, but specifically lean body mass may be affected by a health-conscious diet in early childhood.

We observed less consistent associations between dietary patterns and later body fat or fat distribution. Although in our population adherence to a ‘Western’ dietary pattern was associated with increased FMI in crude models, this association was explained by sociodemographic and lifestyle factors. The only patterns associated with later body fat after adjustment for confounders were the RRR-derived patterns, constructed on the basis of variation in body composition. The first RRR pattern—which was characterized by high intake of refined grains, meat, potatoes, fish, soups and sauces, and sugar-containing beverages,—was associated with a higher fat mass index, a higher body fat percentage, and a higher android/gynoid fat ratio. The second RRR pattern, characterized by intake of whole grains, pasta and rice, and vegetable oils, was associated with a lower body fat percentage, but was associated with a lower FMI only after additional adjustment for BMI-SDS at 1 year.

In contrast to several previous studies [5, 9], but in line with one other study [10], we used reduced-rank regression as exploratory approach to identify which dietary patterns in early childhood explain most variation in body composition. We chose to use body composition measures as response variables, because we were interested in exploring which patterns best predict body composition. The dietary patterns identified with this approach can be used as starting point for subsequent studies and for development of new dietary guidelines for young children to prevent adiposity [11]. Additionally, patterns based on variation in FMI and FFMI can be used in future studies to evaluate the relation between diet and other health outcomes, as body composition is a possible intermediate factor in many diet-disease associations.

### Strengths and limitations

An important strength of our study is that we had a prospective study design with detailed information available on a large number of potential confounders. Previously, several family sociodemographic and lifestyle characteristics have been related to child dietary patterns [18], and to child body composition [35].These factors are thus important to take into account when studying the relation between diet and body composition. Previous studies were not always able to adjust for important factors such as parental BMI and lifestyle. Furthermore, of all children in our study population with information on food intake more than 80 % participated in the body composition measurements at the age of 6 years.

A limitation of our study is that we measured food intake with an FFQ, which is known to be prone to measurement error. However, an FFQ measures habitual diet rather than diet on just one or a few days, and is considered appropriate to use for dietary pattern analysis [36]. Furthermore, we adjusted our analyses for total energy intake to reduce measurement error [37]. A strength is that we used several approaches to examine dietary patterns, as there is currently not one best approach to study overall diet. With the diet score we could examine the effect of adhering to current dietary guidelines on body composition, the PCA-derived patterns show the existing dietary patterns and interrelationships between intake of different food groups in our study population, and with the RRR-derived patterns we could examine which patterns predict body composition best [8]. A limitation is that we only had information available on dietary intake at the age of 1 year. A small proportion of the children in our study (n = 386) were still transitioning from an infant diet based on breast or formula milk to a varied table food diet. In sensitivity analyses, we observed that excluding these children attenuated the results on one of the patterns, and it would thus have been valuable to reassess the associations with diet measured at a later point in time.

A major strength of our study is that we performed detailed measurements of child body composition using DXA. Many previous studies assessed body composition based on total body weight, while in our study we were able to distinguish between fat mass and fat-free mass. However, how to best define body composition indices in children remains an area of debate since these can be highly dependent of height [38]. Furthermore, childhood height in itself has also been associated with obesity later in life [39]. However, as sensitivity analyses, we also analyzed associations of all dietary patterns with absolute fat mass and fat-free mass with and without adjustment for height, which resulted in similar results as obtained for FMI and FFMI, suggesting that the association between dietary patterns and body composition may be independent of the child height.

## Conclusion

Dietary patterns characterized by high intake of fruit, vegetables, grains, and vegetable oils at the age of 1 year, were associated with a higher fat-free mass index at the age of 6 years. Using reduced-rank regression we additionally identified a pattern that predicted a higher fat and fat-free mass index and a higher body fat percentage, which was characterized by high intake of refined grains, meat, potatoes, fish, soups and sauces, and sugar-containing beverages. These findings may aid in developing dietary guidelines for young children. Future studies should explore whether these differences in body composition track into later life and whether these differences are independent of later dietary patterns.

## Electronic supplementary material

Below is the link to the electronic supplementary material.

Supplementary material 1 (PDF 387 kb)

## Abbreviations

BMI: Body mass index
BF%: Body fat percentage
DXA: Dual-energy X-ray absorptiometry
FFQ: Food frequency questionnaire
FFMI: Fat-free mass index
FMI: Fat mass index
PCA: Principal component analysis
RRR: Reduced rank regression
