# Supplementary material for: A priori and a posteriori dietary patterns at the age of 1 year and body composition at the age of 6 years: the Generation R Study
Source: Eur J Epidemiol. 2016 Jul 6;31(8):775–83. doi: 10.1007/s10654-016-0179-x (PMC5005385; doi:10.1007/s10654-016-0179-x)
Supplement: Supplementary file 1 — Supplementary material 1 (PDF 387 kb) [file 10654_2016_179_MOESM1_ESM.pdf]

SUPPLEMENTARY MATERIAL

A priori and *a posteriori* dietary patterns at the age of 1 year and body composition at the age of 6 years: The Generation R Study

Trudy Voortman, Elisabeth T.M. Leermakers, Oscar H. Franco, Vincent W. V. Jaddoe, Henriette A. Moll, Albert Hofman, Edith H. van den Hooven, Jessica C. Kiefte-de Jong

Correspondence and reprint requests: Trudy Voortman, Department of Epidemiology, Erasmus MC; Office Na-2909, PO Box 2040, 3000 CA Rotterdam, the Netherlands
Phone: +31 10 70 44214; Fax: +31 10 70 44657; email: trudy.voortman@erasmusmc.nl

Supplementary Table 1. Characteristics of the children and their parents before and after multiple imputation (*n* = 2,026)

	N	Original data[1]	Imputed data[1]
**Parental characteristics**			
Maternal age (y)	2,026	32.3 (22.9 – 39.9)	*No missings*
Maternal BMI at enrollment (kg/m^2)	1,849	23.3 (18.9 – 34.8)	23.3 (18.9 – 34.8)
Parity			
0	1,242	1,242 (63.0%)	1,271 (62.7%)
1	557	557 (28.2%)	575 (28.4%)
≥ 2	173	173 (8.8%)	180 (8.9%)
Folic acid supplement use (%)			
Started periconceptional	970	970 (63.5%)	1,252 (62.7%)
Started in first 10 weeks	448	448 (29.3%)	597 (29.5%)
Never	110	110 (7.2%)	177 (8.9%)
Alcohol use during pregnancy (%)			
Never	517	517 (30.8%)	619 (30.6%)
Until pregnancy was known	278	278 (16.6%)	343 (16.9%)
Continued	881	881 (52.6%)	1,064 (52.5%)
Smoking during pregnancy (%)			
Never	1,466	1,466 (79.5%)	1,596 (78.8%)
Until pregnancy was known	186	186 (10.1%)	213 (10.5%)
Continued	193	193 (10.5%)	217 (10.7%)
Paternal smoking (%)			
No	947	947 (61.7%)	1,233 (60.9%)
Yes	589	589 (38.3%)	793 (39.1%)
Paternal education (%)			
Primary or secondary school	542	542 (29.0%)	630 (31.1%)
Higher education	1,328	1,328 (71.0%)	1,396 (68.9%)
Net household income per month (%)			
<2200 €	359	359 (20.3%)	448 (22.1%)
≥2200 €	1,413	1,413 (79.7%)	1,578 (77.9%)
**Child characteristics**			
Gender (%)			
Boys	1,002	1,002 (49.5%)	*No missings*
Girls	1,024	1,024 (50.5%)	*No missings*
Breastfeeding (%)			
Exclusive for at least 4 months	552	552 (30.2%)	605 (29.9%)
Partial in the first 4 months	1,101	1,101 (60.2%)	1,211 (59.8%)
Never	176	176 (9.6%)	210 (10.4%)
Introduction complementary feeding (%)			
After 6 months	800	800 (39.6%)	803 (39.6%)
3-6 months	1,136	1,136 (56.3%)	1,139 (56.2%)
0-3 months	83	83 (4.1%)	84 (4.2%)
Television watching at 2 years (h/day)	1915	0.9 (0 – 2)	0.9 (0 – 2)
Child age at FFQ (mo)	2,026	12.9 (12.2 – 19.2)	*No missings*
Total energy intake (kcal/d)	2,026	1267 (737 – 2080)	*No missings*
Child age at center visit (y)	2,026	5.9 (5.7 – 6.5)	*No missings*

[1]*Values are valid percentages for categorical variables or medians (95% range) for continuous variables.*

Supplementary Table 2. Multivariable-adjusted associations of dietary patterns at 1 year of age with additional growth and body composition outcomes at 6 years of age.

	**Weight (SDS)** ***n* =2,026**	**Height (SDS)** ***n* = 2,026**	**BMI (SDS)** ***n* = 2,026**	**BF% (SDS)** ***n* = 1,980**	**A/G ratio (SDS)** ***n* = 1,980**
	β (95%CI)	β (95%CI)	β (95%CI)	β (95%CI)	β (95%CI)
**Diet score**					
Per SD	**0.06 (0.03; 0.10)****	**0.05 (0.01; 0.09)***	**0.06 (0.02; 0.09)****	0.00 (-0.03; 0.03)	-0.00 (-0.04; 0.04)
Q1	*Reference*	*Reference*	*Reference*	*Reference*	*Reference*
Q2	0.04 (-0.05; 0.13)	-0.01 (-0.13; 0.10)	0.07 (-0.02; 0.16)	0.01 (-0.08; 0.10)	-0.04 (-0.14; 0.06)
Q3	0.04(-0.05; 0.14)	-0.02 (-0.14; 0.09)	0.07 (-0.02; 0.16)	-0.05 (-0.14; 0.04)	-0.06 (-0.16; 0.04)
Q4	**0.19 (0.10; 0.29)****	**0.15 (0.03;0.27)***	**0.18 (0.08; 0.27)****	0.02 (-0.08; 0.11)	0.01 (-0.10; 0.11)
**PCA Health-conscious pattern**					
Per SD	0.03 (-0.01; 0.07)	0.01 (-0.04; 0.05)	**0.04 (0.01; 0.08)***	-0.01 (-0.04; 0.03)	-0.01 (-0.05; 0.03)
Q1	*Reference*	*Reference*	*Reference*	*Reference*	*Reference*
Q2	0.04 (-0.06; 0.13)	0.05 (-0.07; 0.16)	0.03 (-0.07; 0.12)	0.01 (-0.09; 0.10)	0.02 (-0.08; 0.12)
Q3	**0.12 (0.02;0.21)***	0.11 (-0.01; 0.22)	**0.10 (0.01; 0.19)***	-0.01 (-0.11; 0.08)	0.02 (-0.08; 0.12)
Q4	**0.13 (0.03;0.23)****	0.08 (-0.04; 0.20)	**0.14 (0.05; 0.24)****	-0.01 (-0.11; 0.08)	-0.02 (-0.12; 0.09)
**PCA Western pattern**					
Per SD	0.00 (-0.04; 0.04)	0.00 (-0.05; 0.05)	0.00 (-0.04; 0.05)	-0.01 (-0.05; 0.04)	0.01 (-0.04; 0.06)
Q1	*Reference*	*Reference*	*Reference*	*Reference*	*Reference*
Q2	-0.01 (-0.11; 0.08)	-0.05 (-0.16; 0.07)	0.02 (-0.07; 0.11)	0.05 (-0.05; 0.14)	0.08 (-0.03; 0.18)
Q3	**0.11 (0.01; 0.20)***	0.04 (-0.07; 0.16)	**0.12 (0.02; 0.21)***	0.03 (-0.07; 0.13)	0.08 (-0.02; 0.19)
Q4	0.03 (-0.08; 0.14)	0.02 (-0.11; 0.15)	0.04 (-0.07; 0.14)	-0.02 (-0.13; 0.09)	0.05 (-0.07; 0.17)
**RRR pattern 1**					
Per SD	**0.10 (0.06; 0.14)****	**0.05 (0.00; 0.10)***	**0.11 (0.07; 0.15)****	**0.08 (0.04; 0.12)****	**0.07 (0.02; 0.11)****
Q1	*Reference*	*Reference*	*Reference*	*Reference*	*Reference*
Q2	**0.09 (0.00;0.19)***	0.04 (-0.08; 0.15)	**0.12 (0.03; 0.21)****	**0.10 (0.01; 0.19)***	0.08 (-0.02; 0.18)
Q3	**0.14 (0.05;0.24)****	**0.12 (0.00; 0.24)***	**0.12 (0.03; 0.21)****	0.08 (-0.01; 0.18)	0.06 (-0.04; 0.16)
Q4	**0.23 (0.13;0.33)****	**0.13 (0.01; 0.25)***	**0.25 (0.16; 0.35)****	**0.14 (0.04; 0.24)****	**0.14 (0.03; 0.25)****
**RRR pattern 2**					
Per SD	0.02 (-0.02; 0.06)	0.02 (-0.03; 0.07)	0.02 (-0.02; 0.06)	**-0.05 (-0.09;-0.01)****	**-0.05 (-0.09;-0.00)***
Q1	*Reference*	*Reference*	*Reference*	*Reference*	*Reference*
Q2	-0.06 (-0.15; 0.04)	-0.03 (-0.15; 0.09)	-0.06 (-0.15; 0.03)	-0.07 (-0.16; 0.03)	-0.07 (-0.17; 0.03)
Q3	**0.11 (0.01; 0.21)***	**0.13 (0.01; 0.25)***	0.07 (-0.03; 0.16)	-0.06 (-0.16; 0.04)	-0.03 (-0.14; 0.08)
Q4	0.04 (-0.07; 0.15)	0.01 (-0.12; 0.15)	0.06 (-0.04; 0.17)	**-0.12 (-0.23; -0.02)***	-0.12 (-0.23; 0.00)

Values are regression coefficients that reflect the difference in outcome (age- and sex-adjusted SD scores) per 1 SD increase in exposure and for quartiles of exposure compared to the lowest quartile.
*Models are adjusted for maternal age, BMI at enrollment, parity, folic acid supplement use, smoking and alcohol use during pregnancy; paternal smoking and education; household income; and child sex, breastfeeding in the first four months of life, timing of introduction of complementary feeding, age at dietary measurement, total energy intake at 1 year, and television watching at age 2 years. BF% and A/G ratio are additionally adjusted for child height. *p<0.05, **p<0.01 Abbreviations: BF%, body fat percentage; A/G ratio, android/gynoid ratio; PCA, principal component analyses; RRR, reduced rank regression*

Supplementary Table 3. Associations of dietary patterns at 1 year of age with childhood body composition at 6 years of age: crude, confounder and baseline BMI adjusted models.

	**BMI (SDS)**<br>***n* = 2,026**<br>β (95%CI)	**FMI (SDS)**<br>***n* = 1,980**<br>β (95%CI)	**FFMI (SDS)**<br>***n* = 1,980**<br>β (95%CI)
**Diet quality score (per SD)**			
Crude	**0.05 (0.02; 0.08)****	0.01 (-0.02; 0.04)	**0.07 (0.03; 0.11)****
Covariate adjusted (main model)	**0.06 (0.02; 0.09)****	0.02 (-0.01; 0.05)	**0.06 (0.02; 0.10)****
Additionally adjusted for baseline BMI	**0.04 (0.01; 0.07)***	0.01 (-0.02; 0.04)	**0.05 (0.01; 0.08)***
**Health-conscious pattern (PCA) (per SD)**			
Crude	**0.04 (0.00; 0.07)***	0.00 (-0.03; 0.04)	**0.05 (0.01; 0.09)***
Covariate adjusted (main model)	**0.04 (0.01; 0.08)***	0.01 (-0.03; 0.04)	**0.05 (0.01; 0.09)***
Additionally adjusted for baseline BMI	0.03 (-0.00; 0.07)	0.00 (-0.03; 0.03)	**0.04 (0.00; 0.08)***
**Western pattern (PCA) (per SD)**			
Crude	0.04 (-0.01; 0.08)	**0.05 (0.01; 0.09)***	0.00 (-0.04; 0.05)
Covariate adjusted (main model)	0.00 (-0.04; 0.05)	-0.01 (-0.05; 0.03)	0.02 (-0.04; 0.07)
Additionally adjusted for baseline BMI	0.01 (-0.03; 0.05)	-0.01 (-0.04; 0.03)	0.02 (-0.03; 0.07)
**RRR pattern 1 (per SD)**			
Crude	**0.14 (0.10; 0.18)****	**0.15 (0.11; 0.19)****	**0.08 (0.03; 0.13)****
Covariate adjusted (main model)	**0.11 (0.07; 0.15)****	**0.10 (0.06; 0.13)****	**0.09 (0.04; 0.14)****
Additionally adjusted for baseline BMI	**0.10 (0.06; 0.14)****	**0.09 (0.06; 0.13)****	**0.08 (0.03; 0.12)****
**RRR pattern 2 (per SD)**			
Crude	0.00 (-0.04; 0.04)	**-0.06 (-0.10; -0.03)****	**0.08 (0.03; 0.12)****
Covariate adjusted (main model)	0.02 (-0.02; 0.06)	-0.03 (-0.07; 0.00)	**0.07 (0.02; 0.11)****
Additionally adjusted for baseline BMI	0.01 (-0.03; 0.04)	**-0.04 (-0.07; -0.01)***	**0.06 (0.01; 0.10)****

Values are regression coefficients that reflect the difference in outcome (age- and sex-adjusted SD scores) per 1 SD increase in exposure, based on imputed data.

Crude models are adjusted for child sex, age at dietary measurement and total energy intake at 1 year.

Covariate models are additionally adjusted for maternal age, BMI at enrollment, parity, folic acid supplement use, smoking and alcohol use during pregnancy; paternal smoking and education; household income; and child breastfeeding in the first four months of life, timing of introduction of complementary feeding, and television watching at age 2 years.

Baseline BMI adjusted models are additionally adjusted for BMI-SDS at the age of 1 year

Abbreviations: BMI, body mass index; FMI, fat mass index; FFMI, fat-free mass index; PCA, principal component analyses; RRR, reduced rank regression

**p<0.05, **p<0.01*

Supplementary Table 4. Associations of dietary patterns at 1 year of age with childhood body composition at 6 years of age in the total population vs. in weaned children only

	**FMI (SDS)**<br>***n* = 1,980 or 1,609**<br>β (95%CI)	**FFMI (SDS)**<br>***n* = 1,980 or 1,609**<br>β (95%CI)
**Diet quality score (per SD)**		
Total population	0.02 (-0.01; 0.05)	**0.06 (0.02; 0.10)****
Weaned children only	0.03 (-0.01; 0.06)	**0.05 (0.00; 0.09)***
**Health-conscious pattern (PCA) (per SD)**		
Total population	0.01 (-0.03; 0.04)	**0.05 (0.01; 0.09)***
Weaned children only	-0.00 (-0.04; 0.04)	0.03 (-0.02; 0.08)
**Western pattern (PCA) (per SD)**		
Total population	-0.01 (-0.05; 0.03)	0.02 (-0.04; 0.07)
Weaned children only	-0.04 (-0.09; 0.01)	0.00 (-0.06; 0.06)
**RRR pattern 1 (per SD)**		
Total population	**0.10 (0.06; 0.13)****	**0.09 (0.04; 0.14)****
Weaned children only	**0.08 (0.04; 0.12)****	**0.07 (0.01; 0.12)***
**RRR pattern 2 (per SD)**		
Total population	-0.03 (-0.07; 0.00)	**0.07 (0.02; 0.11)****
Weaned children only	-0.03 (-0.07; 0.01)	0.05 (-0.00; 0.10)

Values are regression coefficients that reflect the difference in outcome (age- and sex-adjusted SD scores) per 1 SD increase in exposure, based on imputed data.

Weaned children were defined as children who no longer receive breast milk or a substantial amount of infant formula (i.e., more than 500 kcal/d).

Models are adjusted for child sex, age at dietary measurement and total energy intake at 1 year and maternal age, BMI at enrollment, parity, folic acid supplement use, smoking and alcohol use during pregnancy; paternal smoking and education; household income; and child breastfeeding in the first four months of life, timing of introduction of complementary feeding, and television watching at age 2 years.

Abbreviations: FMI, fat mass index; FFMI, fat-free mass index; PCA, principal component analyses; RRR, reduced rank regression

**p<0.05, **p<0.01*
